# Purpura

**Published:** 1910-06

**Authors:** L. C. Allen

**Affiliations:** Hoschton, Ga.


### PURPURA.

BY L. C. ALLEN, M. D., HOSCHTON, GA.

Purpura is a symptom often met with in general practice, and not infrequently indicates that the condition is a grave one. The subject has not received the attention from the profession that its importance deserves. Under the name purpura a large numbr of symptoms, or diseases, which have the common symptom of extravasation of blood under the skin, or mucous membrane, are described. In studying the literature of this subject what most impresses one is the chaotic condition in which this literature is found. Our knowledge of hæmorrhagic conditions is, as yet, far from satisfactory. The direct, immediate cause of the hæmorrhages we do not know. For instance, we do not as yet know why we should have subcutaneous bleeding in one case of rheumatism, or malaria, and not in another. The exact pathological changes that are present in the blood, or blood vessels, in purpuric conditions need further elucidation.

Purpura is doubtless an eliminative reaction of the system to infection, or some form of toxæmia. This is a modern view. Accepting this view as correct, purpura may be classified, pathologically considered, as follows:

1. *Infectious Purpura.* This is found in typhoid fever, pneumonia, the exanthenata, septicæmia, pyæmia, malaria, rheumatism, and other infectious diseases.

2. *Toxic Purpura.* After the administration of certain drugs, such, for instance, as quinine, copaiba, mercury, ergot, the iodides, and chloral hydrate, a purpuric eruption is sometimes seen.

3. *Cachetic Purpura.* This form is seen in cases of cancer, tuberculosis, Bright's disease, Hodgkin's disease, leucæmia, hæmophilia, and the debility of old age.

4. *Neurotic Purpura.* This form is met with in hysteria, after a fright, or profound emotion, and in various other nervous abnormalities.

Clinically considered, purpura is commonly divided into two varieties: Purpura Simplex and Purpura Hæmorrhagica.

In purpura simplex the hemorrhages occur beneath the skin. This form is most commonly met with in children. There is more or less malaise, loss of appetite, sometimes pain in the joints, and occasionally a diarrhœa.

The prognosis is favorable. The patient usually gets well in a week or ten days.

In purpura hæmorrhagica, in addition to the subcutaneous hæmorrhages, there is bleeding from the mucous membranes, such as the nose, kidneys, stomach, and other places. This bleeding comes on in the form of an oozing from the mucous membrane. It is most frequently seen during the course of typhoid fever, malaria, the exanthemata, rheumatism, or any serious infection. The purpuric spots vary greatly in size—from the size of a pin-head to that of the palm of the hand. Gangrene of the skin has been known to follow extensive ecchymoses. Severe vomiting is common. Pure blood may be puked up. Internal hæmorrhages into the brain, and its meninges, or into the lungs, or suprarenal capsule, or from the uterus, may occur. The spleen and liver may be enlarged, and jaundice is not uncommon. The gums may be spongy and bleed, but the teeth are never loosened, as in scurvy.

There is a severe type of purpura hæmorrhagica known as *acute* or *fulminating,* in which the prognosis is very grave. In this form 75 per cent. of the patients die. There is usually a chill, with the temperature rising to 103° or 105°, followed with marked prostration, purpuric spots rapidly appearing, and with bleeding from mucous membranes. Restlessness, delirium, and stupor supervene, and the patient succumbs to hæmorrhage, or dies in a condition of coma within a few days. Hæmorrhages into the brain, suprarenal capsule, or other viscera may prove quickly fatal. Purpura occurring in the aged, or in the cachectic, is an ill omen. Purpura is a finger that points to death when it comes on in a case of chronic renal, hepatic or malignant disease. Occurring in children, or those in middle life, during the course of an infectious disease, the outlook is not so bad.

In the *treatment* of purpura there are two remedies that I especially recommend, viz.: the suprarenal extract, and the chloride of calcium. According to my experience these are by far the best remedies we have for this condition. Aromatic sulphuric acid is probably useful, and turpentine is highly recommended by some writers. Astringents, such as alum, lead, tannic and gallic acid, are also recommended by writers. I have little faith in them, except in cases where they may be used locally. Tannic, or gallic acid, or remedies containing them, like hydrastis, might prove beneficial in cases of hæmorrhage from the kidneys, or alimentary canal. Ergot is another remedy that may prove valuable.

A word of caution as to the diet. These patients should never be allowed alcohol in any form. All food and drink should be taken in a cool state, and in small quantities. Milk is generally regarded as the most suitable food.

The following case will serve to illustrate a form of purpura often met with, and also the treatment to be employed. Mr. B., aged 30, single, school teacher, had a moderate case of typhoid fever. Progressed very well up to middle of third week; temperature ranging from 101 to 103, pulse 100 to 110, some diarrhœa, but easily controlled, tympanitis moderate, some nausea. No delirium. About the 18th day he began to spit up small amounts of blood, but the amount was so small that no special attention was given to it. On the next day this was noticed to be increasing in amount, but still not sufficient in quantity to cause us to think that it required any special treatment. On the third day, however, I was sent for early in the morning, and on arrival, found that he had vomited up blood in large amounts several times during the night; both nostrils were bleeding slowly, but constantly; he was still spitting out blood, and now every few minutes; he was also passing considerable blood from the kidneys; and purpuric spots about as large as a grain of corn had occurred all over his body. His pulse had run up to 160, and were very, very weak; temperature subnormal; still in his right mind, but very much alarmed, and, as I thought, with good cause. Fortunately, the bowels were quiet.

The condition of this patient appeared to be desperate. I proceeded as follows: I first gave him a hypodermic of morphine and atrophine, for its stimulating and quieting effect, and because I did not want the bowels to move. Then I took a mixture of equal parts of normal salt solution, warm, and adrenalin chloride solution, 1 to 1000, and with a cotton applicator, made a thorough application to the entire mucous membrane of both nasal cavities. I instructed nurse how to do this, and directed it to be repeated every 3 hours regularly, and oftener if hæmorrhages recurred. This stopped the nasal bleeding at once. I want to call attention to the point that I would have given the adrenalin internally, but I knew that in making the application to such an extensive area of mucous membrane that enough of it would be absorbed to produce decided constitutional effects. This proved to be the case, for it was easy to discover a marked effect upon the pulse in a very few minutes. Next I gave him 10 grains of Chloride of Calcium, in the form of an elixir, and directed this to be repeated every three hours. Also gave him strychnine and digitalis for the heart. That was all the first day. He vomited blood once more. Never had any return of the nasal hæmorrhage, but the adrenalin applications were kept up for three or four days, when they were left off, and the remedy was then given internally. He continued to pass bloody urine for three or four days, but that was the only loss of blood he sustained. He had a little fever, at irregular intervals, for about ten more days, when it finally left him. Strychnine and digitalis were discontinued after a few days, but the calcium was continued until convalescence was well established The recovery was uneventful.

### GRIPPE.

In the treatment of grippe it is most important to bear in mind its very obstinate character and the necessity of promptly and thoroughly expelling the causes of the disease from the system, thus preventing serious complications and dangerous sequelae.

Tongaline is particularly indicated in grippe on account of its pronounced stimulating action on the liver, the kidneys, the intestines, the pores asd the circulation, thus inducing general elimination.

Furthermore, because the effects of Tongaline are so fully equalized there is no disturbance of any particular organ, but a speedy return to normal conditions.

### HIGHEST THERAPEUTICAL VALUE.

Dioviburnia has stood the critical test of the most exacting physicians for years and has been pronounced of the highest therapeutical value. Can always be relied upon in all functional disorders of the uterus and appendages, whether acute, subacute or chronic.

